# The Impact of the Predictive Nursing Education Process on Degree of Comfort and Quality of Life for Patients in the Oncology Department

**Published:** 2017-09

**Authors:** Yan YU, Lijuan HU, Xingu CHEN, Mei GE, Huijuan ZHU, Yusheng YAN

**Affiliations:** 1. Dept. of Cardio-Thoracic Surgery, Zhujiang Hospital of Southern Medical University, Guangzhou, Guangdong, P.R. China; 2. Dept. of Oncology, Zhujiang Hospital of Southern Medical University, Guangzhou, Guangdong, P.R. China; 3. Dept. of Hepatobiliary Surgery, Zhujiang Hospital of Southern Medical University, Guangzhou, Guangdong, P.R. China; 4. Dept. of General Surgery, Zhujiang Hospital of Southern Medical University, Guangzhou, Guangdong, P.R. China

**Keywords:** Predictive nursing, Cancer, Degree of comfort, Quality of life

## Abstract

**Background:**

We investigated the application of a predictive nursing education process on the degree of comfort and quality of life for cancer patients.

**Methods:**

A total of 168-cancer patient in Zhujiang Hospital of Southern Medical University, Guangzhou, Guangdong, China between June 2014 and June 2016 were enrolled and admitted for surgery or radiotherapy/chemotherapy treatment. Patients were randomly divided into control and observation groups, each containing 84 cases. Patients in the control group received routine cancer care, whereas the observation group received care incorporating a predictive nursing education process. Patients were assessed after admission and prior to discharge via the Kolcaba’s comfort status scale (through a General Comfort Questionnaire (GCQ)), quality of life scale (QOL), and Barthel Index (BI) for activities of daily living (ADL). Patient mental state was also evaluated using the Hamilton Anxiety Rating Scale (HAMA) and Hamilton Depression Rating Scale (HAMD).

**Results:**

GCQ, QOL, and BI scores of the observation group were all significantly higher than those of the control group (*P* <0.05). HAMA and HAMD scores were significantly lower in the observation group compared to the control group (*P* < 0.05).

**Conclusion:**

The predictive nursing education process could significantly improve degree of comfort and activities of daily living for cancer patients, and thus improve mental states and quality of life.

## Introduction

Cancer is currently the disease with highest morbidity and mortality rates worldwide, placing heavy economic burdens and mental strain (1) on patients, families, and society. The occurrence and progression of cancer can seriously impair patient physical and mental functions. A variety of clinical treatments such as surgery, radiotherapy, and chemotherapy inhibit tumor growth, but also increase patient physical and mental suffering, and often become risk factors for quality of life (2). Quality nursing services play a positive role in reducing patient physical and mental discomfort during hospitalization, thus improving the treatment effect (3).

The predictive nursing education process starts from the promise of evidence-based medicine, summarizing characteristics of different diseases and pain behaviors of patients within the department. It is guided by a new type of medical model comprised of comprehensive intervention regarding the social, psychological, and physical well-being of patients. The integrative management of admission inquiry, treatment records during hospitalization, and follow-up after discharge is applied to minimize various adverse factors derived from both the tumor and the treatment. The education process is intended to improve the quality of nursing services and cancer patient survival satisfaction (4, 5).

In this study, the predictive nursing education process was applied to cancer patients at Zhujiang Hospital.

## Materials and Methods

### Subjects

A total of 168 cancer patients admitted to Zhujiang Hospital of Southern Medical University, Guangzhou, Guangdong, China between June 2014 and June 2016 who were treated with surgery, radiotherapy, or chemotherapy were selected. Selected patients were required to meet the following inclusive criteria: [1] aged 18 to 75, [2] receiving cancer treatment for the first time, [3] exhibit good treatment compliance, [4] possess complete clinical records, and [5] provide informed consent. Subjects were excluded based on the following conditions: [1] could not be properly evaluated in a variety of scale assessments due to primary neurological and psychiatric disorders, [2] suffering from other organ dysfunctions affecting cancer treatment, [3] having other non-medical issues during treatment that seriously affected treatment, nursing work, and impact evaluation.

Selected subjects were randomly divided into control and observation groups, with 84 patients in each. The control group included 46 males and 38 females with an average age of 48.6 ± 12.3 and an average education time of 18.9 ± 5.5 years. In this group, there were 35 cases of lung cancer, 21 cases of liver cancer, 9 cases of gastric cancer, 10 cases of colorectal cancer, 7 cases of breast or cervical cancer, and 2 cases of other cancers. Based on clinical TNM staging, 25 cases were stages I or II, 47 were stage III, and 12 were stage IV. Regarding treatment, 13 cases underwent surgery only, 46 cases underwent surgery combined with radiotherapy and chemotherapy, and 25 cases received radiotherapy and chemotherapy only. The observation group included 50 males and 34 females with an average age of 47.7 ± 14.5 and an average education time of 17.6 ± 5.3 years. In this group, there were 37 cases of lung cancer, 20 cases of liver cancer, 10 cases of gastric cancer, 8 cases of colorectal cancer, 6 cases of breast or cervical cancer, and 3 cases of other cancers. Based on clinical TNM staging, there were 22 stages I or II cases, 47 stage III cases, and 15 stage IV cases. Regarding treatment, 12 cases underwent surgery only, 45 cases underwent surgery combined with radiotherapy and chemotherapy, and 27 cases received radiotherapy and chemotherapy only. Patient data was comparable between the two groups.

The study was approved by Ethics Committee of the university and informed consent was taken from the subjects before the study.

### Methods

Two teams were involved in this study. The clinical and nursing team completed the study, and the research team conducted all the scale assessments, including instructing patients in questionnaire completion, consolidation of data, and statistical analysis. The control group received routine cancer care, which consisted of health education, psychological counseling, and the establishment of a sympathetic ward environment. Responsible nurses took the initiative to explain possible causes of tumor formation, treatments, precautions, and complications of intervention. Patients were advised in seminars regarding appropriate, balanced diet, and rest in order to cultivate a healthy lifestyle using vivid images, videos, and eye-catching prompts. Psychological counseling entailed encouraging and mobilizing a cooperative enthusiasm from patients and their families. Patient confidence in overcoming the disease was bolstered through peer education or music in order to reduce anxiety, depression, and other negative emotions. A sympathetic ward environment was cultivated by solving difficulties in patient life in a timely manner. In addition, nutritional support was provided during infusion, examination, and perioperative/postsurgical care to reduce the incidence of complications. Responsible nurses conducted a careful daily assessment of patient lifestyles, medication status, and diet. The nursing plan was adjusted regularly based on clinical manifestations and examination results. The head nurse or nurse-in-charge was responsible for evaluation and delegation of directives.

The observation group adopted a predictive nursing education process. First, a nursing education process was developed. A nursing quality control group was established, which included 1 to 2 nurses-in-charge, a head nurse, and 1 to 2 nursing administrative staff. This group was responsible for nursing skills training and assessment, nursing quality evaluation, and proposals for quality improvement. After extensive solicitation of perspectives from health care workers, patients, and their families, clinical and nursing experts developed a detailed, operable, and evaluable nursing education process based on the clinical characteristics of cancer and its treatment strategies. This education process was outlined in a tabular form. The completion of each step was recorded and dated in the table by nurses, creating a convenient method for verification and assessment. The similarities and differences between nursing care for different cancer types was emphasized. The social backgrounds of patients and their families and their different needs were stressed as well for more targeted nursing care.

The nursing staff was managed hierarchically. Junior nurses possessed 1 to 3 years of care experience, and had mastered routine care operations. They were required to report emergency conditions in a timely manner to physicians or supervisors and provide assistance in handling such situations. They were in charge of nursing 1 to 2 low-risk patients. Intermediate nurses had a minimum of 3 years of care experience, and could identity emergency conditions in a timely manner and take appropriate measures. They were in charge of nursing 1 to 2 high-risk patients. Senior nurses or nurses-in-charge had a minimum of 5 years of care experience. They were mainly responsible for caring for postoperative or critically ill patients. Possible complications were to be predicted early so that practical measures could be developed for timely intervention in order to shield patients from negative emotions and provide good conditions for disease treatment and nursing care. A well-organized plan for discharge and follow-up was established. Patients were encouraged to lead a good lifestyle and go to the hospital for regular check-ups. Patients were also advised to correctly follow medical advice regarding medication. Guidance was provided to identify adverse reactions and incidences where patients should return to the hospital for check-up.

### Outcome Measures and Assessment Criteria

Patients were assessed after admission and before discharge using the Kolcaba’s Comfort Status Scale (GCQ), Quality of Life Scale (QOL), and Barthel Index (BI) for activities of daily living (ADL), as well as with the Hamilton Anxiety Rating Scale and Hamilton Depression Rating Scale (HAMA and HAMD respectively) for mental state. The General Comfort Questionnaire (GCQ) was comprised of 28 questions concerning physiological, psychological, spiritual, social/cultural, and environmental aspects. A 4-point Likert Scale was adopted for scoring with 1 indicating strongly disagree and 4 indicating strongly agree. Questions phrased in the negative adopted a scoring scale with 1 indicating strongly agree and 4 indicating strongly disagree. Higher scores therefore indicated increasing patient comfort.

There were 12 topics in QOL questionnaires: food appetite, mental state, sleeping, fatigue, pain, family understanding and cooperation, colleague understanding and cooperation, self-understanding of cancer, attitude towards treatment, daily life, adverse effects and facial expressions. Each item was graded in five broad bands within a 60 point scale: 51 to 60 points indicated good, 41 to 50 points indicated fairly good, 31 to 40 points indicated average, 21 to 30 points indicated poor, and < 20 points indicated very poor.

BI used 10 variables to describe ADL and mobility: feeding, bathing, grooming, dressing, fecal continence, urinary continence, toilet use, transfers (e.g. from chair to bed), walking for 45 meters, and climbing stairs. Each variable was assessed in 4 levels, each rated from 0–10 (15) points: self-care, less help needed, more help needed, and completely dependent. A higher score indicates a better activity of daily living. The overall rating were as follows: > 60 indicated good status, 41–60 indicated average (i.e. some dysfunction and slight dependence), and scores < 40 indicated poor (i.e. more or complete dependence).

Fourteen items were evaluated using HAMA, each scored from 0–4. Higher scores indicated worse symptoms. Overall scores were rated as severe anxiety (≥ 29), significant anxiety (21–28), certain anxiety (14–20), little anxiety (7–13), and no anxiety (≤ 7). HAMD used 24 variables, each scored from 0–4 points. Higher scores indicated more serious symptoms. Overall scores were rated as severe depression (≥35), moderate depression (20–34), mild depression (8–19), and no depression (≤8).

### Statistical Analysis Methodology

SPSS 20.0 software (Chicago, IL, USA) was used for statistical analysis. Measurement data were expressed as mean ± standard deviation. An independent sample *t* test was adopted for comparison between groups, and paired t test was used for comparison within a group. Count data were expressed as the number of cases or the percentage (%). X^2^ test was adopted for comparison between groups. Non-parametric rank sum test was used for grade data. A difference of *P* < 0.05 was statistically significant.

## Results

### Comparison of GCQ Results

The GCQ score in the observation group was significantly higher than that of the control group (*P*<0.05) after nursing care (Table 1).

**Table 1:** GCQ Results Comparison (points)

**Group**	**Before Nursing Care**	**After Nursing Care**	***t***	***P***
**Control Group**	56.9±13.8	70.4±16.6	4.786	0.025
**Observation Group**	55.8±14.5	85.3±13.7	5.328	0.02
***t***	0.192	4.625		
***P***	0.869	0.028		

### Comparison of QOL Results

The percentage of positive cases (good + fairly good) in the observation group was higher than that of the control group (*P*<0.05) after nursing care (Table 2).

**Table 2:** QOL Results Comparison (cases or %)

**Group**	**Total Cases**	**Good Cases**	**Fairly Good Cases**	**Average Cases**	**Poor Cases**	**Very Poor Cases**	**Good + Fairly Good Cases (%)**
**Control Group**	84	29	26	14	10	5	55 (65.5)
**Observation**	84	40	27	9	6	2	67 (79.8)
**Group**							
**Z/X^2^**				−2.16			4.311
***P***				0.031			0.038

### Comparison of BI Results

The BI score in the observation group was significantly higher than that of the control group (*P* < 0.05) after nursing care (Table 3).

**Table 3:** BI Results Comparison

**Group**	**Total Cases**	**Score**	**Good Cases**	**Average Cases**	**Poor Cases**
**Control Group**	84	57.6±7.8	48	29	7
**Observation Group**	84	65.8±9.2	65	15	4
**Z/t**		5.532		−2.731	
***P***		0.014		0.006	

### Comparison of HAMA and HAMD Scores

The HAMA and HAMD scores of the observation group were significantly lower than those of the control group (*P*<0.05) after nursing care (Table 4).

**Table 4:** HAMA and HAMD Results Comparison (points)

**Group**	**HAMA**		**HAMD**	
	Before Nursing	After Nursing	Before Nursing	After Nursing
**Control Group**	25.6±6.7	16.7±5.4	32.5±5.9	17.8±4.3
**Observation Group**	26.2±6.6	10.8±5.2	33.4±6.2	12.3±4.2
***t***	0.156	5.754	0.132	5.632
***P***	0.921	0.008	0.956	0.011

## Discussion

The predictive nursing education process highlights patient-specific conditions and individual needs. Patients are able to receive continuous care and guidance from nurses with professional skills and insights. Systematic assessment and management were carried out, and proactive nursing services were provided to effectively improve nursing quality (6). The predictive nursing education process adheres to the basic principles of focusing on prevention in combination with early active treatments to better avoid occurrence of complications (7). For example, risk factors for possible pulmonary infection were evaluated and preventive measures were adopted, i.e. regular indoor ventilation, moderate humidity, switching body position every 30 min to 2 h, and mist inhalation. The predictive nursing education process provides patients with specialized, personalized, and more targeted care services, promoting patients recovery (8).

In this study, GCQ, QOL, and BI scores were all significantly improved in the observation group post-nursing care. Correspondingly, HAMA and HAMD scores decreased significantly. This indicated that the predictive nursing education process significantly improved level of comfort, activities of daily living, and quality of life, as well as lifted the spirits of cancer patients. The work attitude and professional conduct of the nurses in charge plays a decisive role in care (9). Cancer patients often lose confidence in life, and their physical and mental functions are severely diminished as a consequence. Due to complications arising from a variety of treatments, patients need good nursing care, and their physical and mental states need to be assessed correctly. In addition, a lack of appropriate self-regulatory ability, accompanied by sleep disorders, pressure ulcers, and emotional loss, etc. can occur in patients (10). Application of scientific, comprehensive, and predictive nursing education processes can stimulate the enthusiasm of the nursing staff to improve treatment effect and quality of life for patients (11, 12).

In summary, the predictive nursing education process consists of the following technical points. The inpatient ward should be maintained at an appropriate temperature, with clean and fresh indoor air, clean and tidy beds, and the maintenance of a high-level of disinfection and ventilation. Patients should maintain comfortable body positions with the use of body position pads if necessary. Pain should be relieved appropriately. The nursing staff should correctly use communication skills and body language to communicate with, fully understand, and care for patients to help eliminate adverse psychological effects. Patients with constipation should be recommended the choice of high-fiber diet, external glycerin enema, or Chinese medicine auricular seed treatment. Patients can get help in case they have difficulties in life.

## Conclusion

The predictive nursing education process could significantly improve the comfort degree, daily living activities of the patients; it can also improve the spirit and quality of life. There is a good value of clinical promotion.

## Ethical considerations

Ethical issues (Including plagiarism, informed consent, misconduct, data fabrication and/or falsification, double publication and/or submission, redundancy, etc.) have been completely observed by the authors.
